# Outcomes of Alcohol Septal Ablation vs Transaortic Septal Myectomy in Elderly Patients With Obstructive Hypertrophic Cardiomyopathy

**DOI:** 10.1016/j.jacadv.2025.102488

**Published:** 2026-01-28

**Authors:** Tedy Sawma, Hartzell V. Schaff, Sina Danesh, Youssef Shahin, Steve R. Ommen, Joseph A. Dearani, Mackram F. Eleid, Jeffrey B. Geske

**Affiliations:** a Department of Cardiovascular Surgery, Mayo Clinic, Rochester, Minnesota, USA; b Department of Cardiovascular Medicine, Mayo Clinic, Rochester, Minnesota, USA

**Keywords:** alcohol septal ablation, ASA, elderly, HCM, hypertrophic cardiomyopathy, older, septal myectomy, septal reduction therapy

Obstructive hypertrophic cardiomyopathy is increasingly recognized in elderly patients.^1^^,^^2^ In patients with obstructive symptoms refractory to maximal medical therapy, septal reduction therapy, whether through alcohol septal ablation (ASA) or surgical septal myectomy, is indicated.^3^ ASA is often preferred for symptomatic patients who are considered to be at increased risk for surgery, and this includes many older patients. However, in high-volume-experienced centers, operative risks of septal myectomy are minimal (<1%) and long-term outcomes are well-established, even among older patients.^2^^,^^4^ There are few studies that compare outcomes of ASA vs myectomy in the elderly.^5^ The present investigation examines short- and long-term outcomes of septal reduction via ASA or septal myectomy in patients 75 years or older.

## Methodology

The Mayo Clinic Institutional Review Board in Rochester, Minnesota, approved the study on November 26, 2024. All patients 75 years or older who underwent first-time ASA or transaortic septal myectomy between 2000 and 2024 were included. Patients with previous septal reduction therapy, those undergoing apical myectomy, and those with severe aortic stenosis or concomitant surgical procedures were excluded. The final study cohort consisted of 281 patients, of whom 119 underwent ASA and 162 myectomy.

Comprehensive resting 2-dimensional transthoracic Doppler echocardiography was performed in all patients preoperatively. Endpoints of the study were long-term survival, trends of residual maximal left ventricular outflow tract (LVOT) gradients, and the need for reintervention by ASA or myectomy.

**Figure 1:**
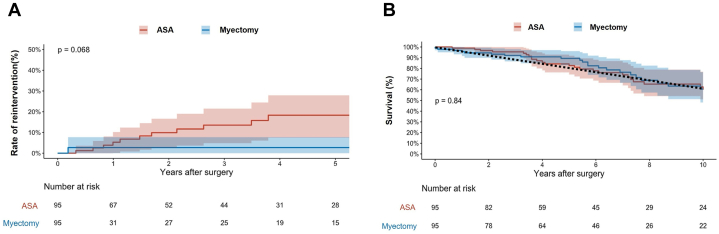
Rate of Reintervention and Long-Term Survival Among Elderly Patients Undergoing ASA vs Septal Myectomy (A) Cumulative incidence curve illustrating the incidence of reintervention among patients undergoing alcohol septal ablation (ASA) vs septal myectomy. (B) Kaplan-Meier curve illustrating long-term survival among patients undergoing ASA vs septal myectomy.

Due to differences in baseline characteristics, propensity scores were generated and used to match patients in a 1:1 ratio. Matching variables included age, sex, body mass index, NYHA functional class, cerebrovascular disease, recent atrial fibrillation, renal failure, diabetes, hypertension, coronary artery disease, beta-blocker use, maximal LVOT gradient at presentation, and ejection fraction (Figure 1A). The Kaplan-Meier method and competing risk analysis were used to generate the curves for long-term survival and the rate of reintervention. To assess trends of residual LVOT gradients, a linear mixed-effect model was used with adjustment for propensity scores. Time was fitted in the model using natural splines to allow for nonlinear effects, and plots were generated with 95% CI. All analyses were performed on R software (version 4.4.2).

## Results

Prior to matching, patients undergoing ASA were older than those undergoing myectomy (79 years vs 77 years; *P* < 0.001) and more likely to be female (78% vs 65%; *P* = 0.015). Following propensity matching, 95 patients undergoing ASA were perfectly matched on all baseline characteristics to 95 patients undergoing myectomy. The median age of the matched group was 78 years (IQR: 76-80) (*P* = 0.4), and 70.5% were females (*P* = 0.62). Median septal thickness was 19 mm (17-22) (*P* = 0.86), and maximal LVOT gradient at presentation was 81 mm Hg (64-107) (*P* = 0.68).

Median volume of injected alcohol was 1.5 mL (1-1.8). Infused coronary arteries included the first septal perforator in 68 patients (35.6%), the second septal perforator in 10 (5.2%), both the first and second in 7 (3.7%), and the third septal perforator in 4 (2.1%).

Operative mortality was 0% in the ASA group and 1% (1 patient) in the myectomy group. Complete heart block necessitating permanent pacemaker implantation developed in 22 patients (23.2%) undergoing ASA and 10 (10.5%) undergoing myectomy (*P* = 0.02).

Survival in both cohorts was comparable to a U.S. age- and sex-matched general population (Figure 1B), and there was no significant difference in survival between the matched groups (*P* = 0.84).

Median maximal LVOT gradient prior to discharge was 25 mm Hg (5-61) in the ASA group and 5 mm Hg (5-9) in the myectomy group (*P* < 0.001). Differences in LVOT gradients persisted in late follow-up (Figure 1C). In the ASA group, 11 patients needed reintervention, 8 through surgical myectomy, and 3 through ASA. In the surgical group, 2 patients underwent repeat septal myectomy. The long-term rate of reintervention was higher in the ASA group (*P* = 0.06) (Figure 1D).

## Discussion

Both ASA and surgical myectomy can be performed with low procedural risk at specialized hypertrophic cardiomyopathy (HCM) centers, regardless of patient age at presentation.^2^^,^^5^^,^^6^ This is reflected by the minimal operative mortality seen in this cohort of obstructive hypertrophic cardiomyopathy patients ≥75 years. Complete heart block requiring pacemaker insertion was 2-fold higher in the ASA group than in the myectomy group, and this is consistent with previous studies.^7^

An important finding of this study is that long-term mortality rates were comparable between the 2 groups and similar to that of a sex- and age-matched population. Notably, in a younger study group, Cui et al reported that while the risk of death is similar over the first 3 years following either ASA or myectomy, survival subsequently favors myectomy.^8^ For elderly patients, such as those in our cohort >75 years, the potential benefit of surgical myectomy on late survival seen in younger patients may be less important than the risk and impact of the procedure on quality of life. This again emphasizes the importance of an experienced multidisciplinary HCM team when evaluating treatment options.

Interestingly, LVOT gradient relief is more complete after septal myectomy than ASA at both early and late follow-up. This is further reflected by the relatively higher rates of reintervention observed in patients undergoing ASA compared to myectomy (*P* = 0.06). Lack of statistical significance likely reflects insufficient statistical power (small sample size).

In conclusion, both ASA and surgical myectomy are safe and effective options for septal reduction therapy in elderly patients with refractory obstructive symptoms. Surgical myectomy provides more complete relief of LVOT gradients but early and long-term clinical outcomes are generally similar with the 2 therapies.

## Funding support and author disclosures

This work has been supported by the Tsai Family. The authors have reported that they have no relationships relevant to the contents of this paper to disclose.
